# Increased Loss Aversion in Unmedicated Patients with Obsessive–Compulsive Disorder

**DOI:** 10.3389/fpsyt.2017.00309

**Published:** 2018-01-15

**Authors:** Kamila E. Sip, Richard Gonzalez, Stephan F. Taylor, Emily R. Stern

**Affiliations:** ^1^Department of Psychiatry, Icahn School of Medicine at Mount Sinai, New York, NY, United States; ^2^Fishberg Department of Neuroscience, Icahn School of Medicine at Mount Sinai, Friedman Brain Institute, New York, NY, United States; ^3^Department of Psychology, University of Michigan, Ann Arbor, MI, United States; ^4^Department of Psychiatry, University of Michigan, Ann Arbor, MI, United States

**Keywords:** decision-making, prospect theory, choice behavior, reward, obsessive–compulsive disorder

## Abstract

**Introduction:**

Obsessive–compulsive disorder (OCD) patients show abnormalities in decision-making and, clinically, appear to show heightened sensitivity to potential negative outcomes. Despite the importance of these cognitive processes in OCD, few studies have examined the disorder within an economic decision-making framework. Here, we investigated loss aversion, a key construct in the prospect theory that describes the tendency for individuals to be more sensitive to potential losses than gains when making decisions.

**Methods:**

Across two study sites, groups of unmedicated OCD patients (*n* = 14), medicated OCD patients (*n* = 29), and healthy controls (*n* = 34) accepted or rejected a series of 50/50 gambles containing varying loss/gain values. Loss aversion was calculated as the ratio of the likelihood of rejecting a gamble with increasing potential losses to the likelihood of accepting a gamble with increasing potential gains. Decision times to accept or reject were also examined and correlated with loss aversion.

**Results:**

Unmedicated OCD patients exhibited significantly more loss aversion compared to medicated OCD or controls, an effect that was replicated across both sites and remained significant even after controlling for OCD symptom severity, trait anxiety, and sex. *Post hoc* analyses further indicated that unmedicated patients’ increased likelihood to reject a gamble as its loss value increased could not be explained solely by greater risk aversion among patients. Unmedicated patients were also slower to accept than reject gambles, effects that were not found in the other two groups. Loss aversion was correlated with decision times in unmedicated patients but not in the other two groups.

**Discussion:**

These data identify abnormalities of decision-making in a subgroup of OCD patients not taking psychotropic medication. The findings help elucidate the cognitive mechanisms of the disorder and suggest that future treatments could aim to target abnormalities of loss/gain processing during decision-making in this population.

## Introduction

Obsessive–compulsive disorder (OCD) is associated with highly distressing fears of negative or bad events (obsessions) and repetitive behaviors aimed at preventing these events and reducing anxiety (compulsions). It has been suggested that a core facet of OCD is impairment of decision-making (1, 2). Experimentally, patients with OCD require more information to arrive at a decision (3–6) and rate themselves as more uncertain (6–8) than healthy volunteers. More recently, models of psychiatric disorders have sought to characterize decision-making impairment within an economic framework such as prospect theory (9–14). In prospect theory, sensitivity to potential negative and positive outcomes (i.e., losses and gains) when making decisions is characterized by a value function that describes the subjective (or experienced) value assigned to monetary losses and gains (15–17). The value function has two main properties. First, the shape of the value function is specified by the formula *x*^α^, where *x* is the monetary value and the value of α determines whether behavior is risk averse (α < 1) or risk seeking (α > 1). The function is typically convex for losses (α > 1, risk seeking) and concave for gains (α < 1, risk averse) (15–17). Second, the subjective (negative) value of a monetary loss (the “disvalue”) is greater than the subjective (positive) value of the equivalent gain—a property known as loss aversion (15–17). Loss aversion is succinctly described by the phrase “losses loom larger” than gains and is measured by the coefficient λ (lambda), which reflects the greater steepness of the value function for losses compared to gains. λ can be interpreted in terms of willingness to accept or reject playing a gamble, so that an individual would require a potential gain to be more than “λ” times larger than a potential loss to accept playing the gamble. For example, an individual with λ = 2 would accept a gamble with 50/50 odds of winning $11 vs. losing $5 but reject a gamble of winning $9 vs. losing $5. Although the seminal work by Tversky and Kahneman (15) provided an initial mean estimate of λ = 2.25 in a group of healthy students, more recent studies on healthy individuals have reported somewhat lower average coefficients typically ranging from 1.2 to 2 (9, 11, 18–26).

Recent work using an economic decision-making framework to study OCD found a value function shape that was indicative of reduced risk aversion in patients compared to healthy controls when making decisions about potential gains (27). Interestingly, this study also found increased probability weighting among OCD patients, which is another core aspect of prospect theory that describes individuals’ tendency to overweight low probability outcomes and underweight high probability outcomes (15–17). By contrast, in a study that looked at risk taking without estimating the value function, Sip et al. (12) found that OCD patients made fewer choices of “risky” gambles having an unknown outcome and more choices of “safe” options having a known outcome compared to healthy controls. Admon et al. (28) had OCD patients and healthy controls play a computer game where their goal was to discard as many “chips” as possible. In the task, chips were allowed to be discarded if they matched a “master chip,” but all discarded chips were hidden from a fictional other player who decided on each trial whether to check if the discarded chips actually did match the master chip and would punish the participant if an unmatched chip was discovered. Safe choices were those where the discarded chip matched the master chip, and risky choices were those where the discarded chip did not match. Similar to the study by Sip et al. (12), results from this study indicated that OCD patients made fewer non-match, risky discards than healthy controls. Although these prior studies have made important contributions to the understanding of abnormal decision-making in OCD, to our knowledge, no prior work has specifically examined loss aversion in this population. The clinical presentation of OCD, characterized by fear of negative events and harm, suggests that there may be an underlying cognitive bias to be more sensitive to potential negative outcomes (i.e., losses) than positive outcomes (i.e., gains), an aspect of behavior that these prior studies were not designed to address. In particular, an investigation of loss processing using a task not directly invoking patient symptoms could elucidate core mechanisms contributing to patients’ hypersensitivity to possible negative events, which could serve as potential targets for novel treatment approaches. In this study, we investigated loss aversion in OCD using a well-established behavioral paradigm where participants accept or reject 50/50 gambles with varying gain/loss values [developed by Tom et al. (25) and utilized by several investigators (9, 11, 20, 22, 26, 29)]. Our sample included groups of medicated and unmedicated OCD patients and healthy control participants across two study sites. We hypothesized that patients would show increased loss aversion compared to controls.

## Materials and Methods

### Participants

We collected samples at two different sites: Site 1 (collected at the Icahn School of Medicine at Mount Sinai) and Site 2 (collected at the University of Michigan). The sample consisted of 43 OCD patients (18 from Site 1 and 25 from Site 2) and 34 HCs (16 from Site 1 and 18 from Site 2). Fourteen OCD patients were unmedicated (uOCD, 5 from Site 1 and 9 from Site 2) and 29 were taking psychoactive medication [medicated OCD (mOCD), 13 from Site 1 and 16 from Site 2] including serotonin-reuptake inhibitors (SRIs) (*n* = 27), lisdexamfetamine (*n* = 1), and clomipramine (*n* = 1). The three final groups (uOCD, mOCD, and HC) were matched for age, education, and sex (see Table 1).

**Table 1:** Demographics and clinical information.

	Unmedicated OCD (uOCD) (*n* = 14)	Medicated OCD (mOCD) (*n* = 29)	HC (*n* = 34)
Age	26.0 (7.2)	26.0 (5.5)	26.7 (7.9)
Education (years)	15.4 (6.9)	16.0 (2.4)	16.2 (2.0)
Sex	11 W, 3 M	14 W, 15 M	20 W, 14 M
Y-BOCS	24.0 (5.8)^a^	19.7 (5.1)	N/A
OCD only current	64.3 (9)	51.7 (15)	0
Comorbid anxiety^b^	35.7 (5)	27.6 (8)	0
Comorbid ICD	7.1 (1)	13.8 (4)	0
Comorbid TD	0	6.9 (2)	0
Comorbid BDD	7.1 (1)	0	0
Comorbid MDD	7.1 (1)	3.4 (1)	0
Depression NOS	0	17.2 (5)	0
Past MDD	42.9 (6)	62.1 (18)	0

*There were no significant differences between uOCD, mOCD, and HC groups in age, years of education, or sex. Cells in the bottom eight rows represent percentage of patient within the group, with the number of patients in parentheses*.

*^a^uOCD patients had significantly higher Y-BOCS scores than medicated OCD patients (*p* = 0.01). For comorbidity counts, patients with multiple disorders contribute to more than one cell; patients with more than one anxiety comorbidity contribute multiple times to the anxiety cell. Chi-square tests revealed no significant differences between uOCD and mOCD patients in proportion of patients displaying the comorbidities listed below (*p* > 0.20 for all)*.

*^b^Includes generalized anxiety disorder (*n* = 6), social phobia (*n* = 3), specific phobia (*n* = 2), and agoraphobia (*n* = 2)*.

*BDD, body dysmorphic disorder; ICD, impulse control disorder; MDD, major depressive disorder; mOCD, medicated obsessive–compulsive disorder; NOS, not otherwise specified; TD, tic disorder; uOCD, unmedicated obsessive–compulsive disorder; Y-BOCS, Yale-Brown Obsessive–Compulsive Scale*.

Participants were assessed for Axis I disorders using structured clinical interviews [for Site 1, using the Mini-International Neuropsychiatric Interview (30); for Site 2, using the Structured Clinical Interview for DSM diagnoses (31)]. HCs were excluded for lifetime diagnosis of Axis I disorder. All OCD patients met DSM-IV criteria for current OCD, excluding primary hoarding subtypes. Patients were excluded for lifetime presence of psychosis, bipolar disorder (by structured clinical interview), and major developmental or neurological disorder (by self-report). Twenty-four patients carried OCD as their only major Axis I diagnosis at the time of assessment (see Table 1 for comorbidities). Symptom severity in OCD was assessed using the Yale-Brown Obsessive–Compulsive Scale (Y-BOCS) (32). All participants gave written informed consent in accordance with the Declaration of Helsinki. The protocol was approved by the institutional review boards at both the Icahn School of Medicine at Mount Sinai and University of Michigan Medical School sites.

### Task

**Figure 1:**
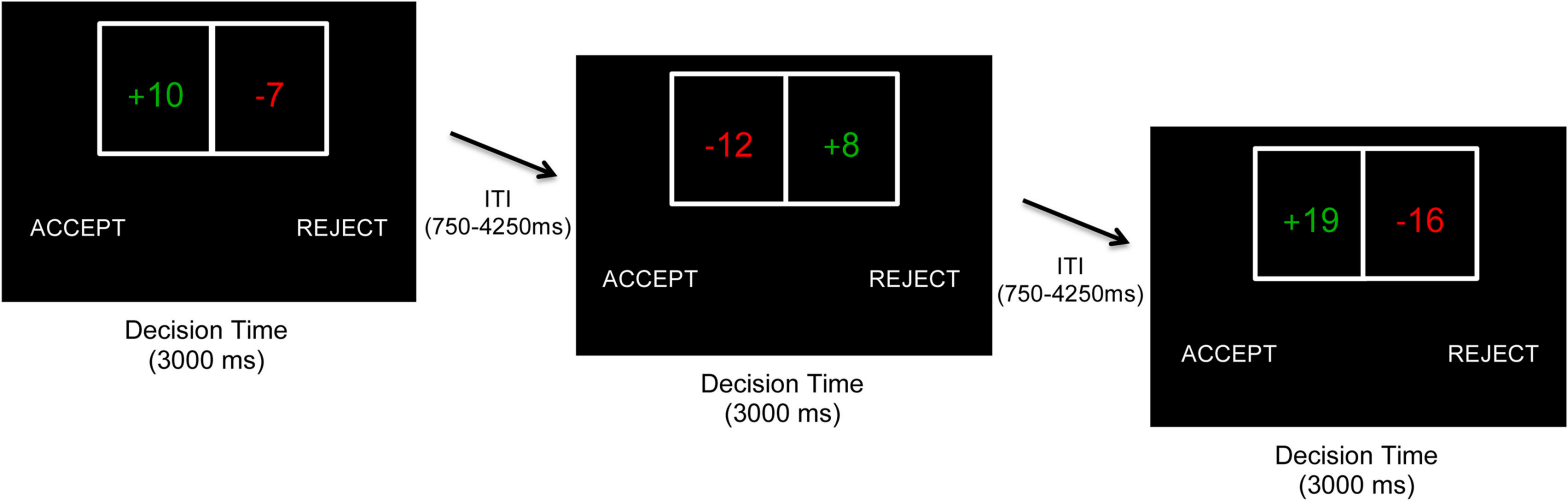
Behavioral task. On each trial, participants decided whether to accept or reject a 50/50 gamble with varying gain/loss values. Gains and losses ranged from $5 to $20 in increments of $1. Gains and losses were presented on the left or right side of the gamble in a pseudorandomized fashion. The side of the screen on which the accept/reject choices were presented were counterbalanced across participants. No immediate outcomes were presented to participants. At Site 1, participants had to make a decision within 3,000 ms (shown). At Site 2 (not shown), participants had to make a decision within 2,500 ms and chose between four choices (accept weakly, accept strongly, reject weakly, and reject strongly), which were collapsed into a binary accept/reject variable for all analyses.

The task was based on that used by Tom et al. (25) and contained 256 trials across 3 runs. On each trial, participants were presented with a gamble showing a 50/50 chance of gaining one amount of money or losing another amount (Figure 1). Possible gains and losses ranged from $5 to $20 in $1 increments, and all possible combinations were presented across the experiment. On each trial, participants decided to accept or reject playing the gamble using button press responses. For half of participants, a decision to accept was made by the left button press (and decision to reject by right button press), whereas for the other half, a decision to accept was made by the right button press (and reject by left button press). Both gain and loss values switched between the left and right sides of the gamble equally throughout the experiment. Outcomes were not presented on each trial; instead, participants were told that one accepted gamble would be randomly selected and played (with a coin toss) at the end of the experiment. Subjects received a real monetary bonus based on participation at the end of the task.

Although the fundamental approach across study sites was the same, there were minor differences in task implementation. First, Site 1 presented the gamble for 3,000 ms, whereas Site 2 presented the gamble for 2,500 ms. Second, Site 1 participants made a binary choice of either accepting or rejecting the gamble, whereas Site 2 participants used four buttons to further qualify their choices based on confidence [accept strongly, accept weakly, reject strongly, and reject weakly; see Ref. (25)]. As in prior studies using this four-response approach (11, 22, 25, 26, 29), this was done to encourage participants to make more deliberate decisions, but data were recoded into a binary accept–reject variable (irrespective of strongly or weakly) for all analyses. Third, participants performed the task in a behavioral testing room for Site 1 and in an fMRI scanner for Site 2 (neuroimaging data to be reported separately). Due to these differences, study site was specified as an additional factor for all analyses.

### Data Analysis

Age and education were compared in two 2 (Site: 1/2) × 3 (Group: uOCD/mOCD/HC) ANOVAs. Sex was compared between groups and study sites using Pearson chi-square. Y-BOCS scores were compared between OCD patient groups with a 2 (Site: 1/2) × 2 (Group: uOCD/mOCD) ANOVA.

The effects of group and study site on percentage of reject choices were analyzed using a 2 (Site: 1/2) × 3 (Group: uOCD/mOCD/HC) ANOVA, with follow-up *t*-tests further interrogating the direction of significant effects. Note that because participants had to choose between rejecting or accepting each gamble, these choices were mutually determined, and there was no need to run an additional analysis of percentage of accept choices (which would provide redundant information). An analysis of decision times [reaction times (RTs)] to make choices was conducted with a 2 (Choice type: accept/reject) × 2 (Site) × 3 (Group) mixed model ANOVA.

To investigate loss aversion, for each participant, a logistic regression analysis (with no intercept) was conducted using gain values (+5 through +20) and loss values (−5 through −20) as predictors of the (log)likelihood of accepting vs. rejecting a gamble. From these subject-level regressions, beta weights were derived reflecting the relationship between increasing losses and the increasing likelihood of rejecting the gamble (β_loss_) and the relationship between increasing gains and increasing likelihood of accepting the gamble (β_gain_). Greater beta values for the loss compared to gain predictor are indicative of loss aversion. The loss aversion coefficient λ was computed as the ratio of β_loss_ to β_gain_, with λ > 1 reflecting greater sensitivity to increasing losses (i.e., greater likelihood to reject) compared to sensitivity to increasing gains (i.e., greater likelihood to accept) (loss aversion), λ < 1 reflecting greater sensitivity to increasing gains than losses, and λ = 1 reflecting equivalent sensitivity to increasing losses and gains. This approach is consistent with that used in the literature (11, 22, 25, 26, 29) and makes the simplifying assumption of equal decision weights of 0.5 for gains and losses and linearity of the value function for the small values used in the study. At the group level, loss aversion coefficients were compared to the reference value of 1.0 (no loss aversion) using one-sample *t*-tests and were compared between the groups using a 2 (Site: 1/2) × 3 (Group: uOCD/mOCD/HC) ANOVA, with follow-up *t*-tests interrogating the direction of significant effects. Given that loss aversion coefficients are frequently positively skewed, we also repeated the above analysis of loss aversion using log(λ) values.

In this report, we do not consider other aspects of prospect theory such as probability weighting and editing. Although the design did not include gain-only or loss-only gambles and thus could not assess risk aversion separately from loss aversion in one model, we did conduct follow-up analyses to disentangle these two effects. For all gambles where gain = |loss| (16 trials, i.e., 50% chance of gaining 5 and 50% chance of losing 5), a risk aversion-only model has gain^α^ = |loss|^α^ (where α is the curvature of the value function) and that equality holds for all α values. For example, a risk aversion-only model would predict that a gamble with a 50% chance of gaining 5 and a 50% chance of losing 5 should be as equally acceptable as a gamble with a 50% chance of gaining 20 and a 50% chance of losing 20, so accept and reject rates should not change as gain/|loss| values increase across gambles where gain = |loss|. By contrast, the loss aversion-only model predicts increased rejection of gambles as gain/|loss| values increase even when gain = |loss|. For example, a loss aversion-only model would predict that a gamble with a 50% chance of gaining 20 and a 50% chance of losing 20 will be less acceptable—rejected more often—than a gamble with a 50% chance of gaining 5 and a 50% chance of losing 5. To test whether a risk aversion-only or loss aversion-only model best fits our data, we ran follow-up logistic regressions using gain/|loss| values (5 through 20) as predictors of the likelihood of rejecting (vs. accepting) a gamble for only those gambles where gain = |loss|. For this analysis, a positive parameter estimate would reflect an increasing likelihood of rejection (decreasing likelihood of acceptance) as gain/|loss| values increase, which, as described above, can be explained by a loss aversion model but not risk aversion model. By contrast, a parameter estimate at or near zero would indicate no relationship between gain/|loss| values and likelihood of rejection and would fit with a risk aversion model but not a loss aversion model. Subject- and group-level data were included in a generalized linear mixed model regression analysis that compared parameter estimates between the groups using the HC group as the reference.

## Results

### Participant Characteristics

Demographic and clinical information are presented in Table 1. There were no significant differences in age between the groups (*F*_2,76_ = 0.1, *p* = 0.89) or study sites (*F*_1,76_ = 2.9, *p* = 0.10) and no interaction between factors (*F*_2,76_ = 1.2, *p* = 0.32). There were also no differences between the groups in years of education (*F*_2,76_ = 0.2, *p* = 0.84) and no interaction between factors (*F*_2,76_ = 1.5, *p* = 0.24), although participants in Site 1 had completed more years of education (16.7) than those in Site 2 (15.3) (*F*_1,76_ = 6.3, *p* = 0.01). The proportion of women to men was not significantly different between groups (χ^2^ = 3.6, *p* = 0.17) or study sites (χ^2^ = 2.1, *p* = 0.15).

There was a main effect of group on Y-BOCS scores: unmedicated OCD patients had significantly greater symptom severity (Y-BOCS score = 24.0) compared to medicated OCD patients (19.7) [*F*_1,42_ = 7.4, *p* = 0.01, Cohen’s d (effect size) = 0.79]. There was no effect of study site (*F*_1,42_ = 0.6, *p* = 0.46) or interaction between group and site (*F*_1,42_ = 1.7, *p* = 0.21) on Y-BOCS scores.

### Percentage and Decision Times for Choices

**Figure 2:**
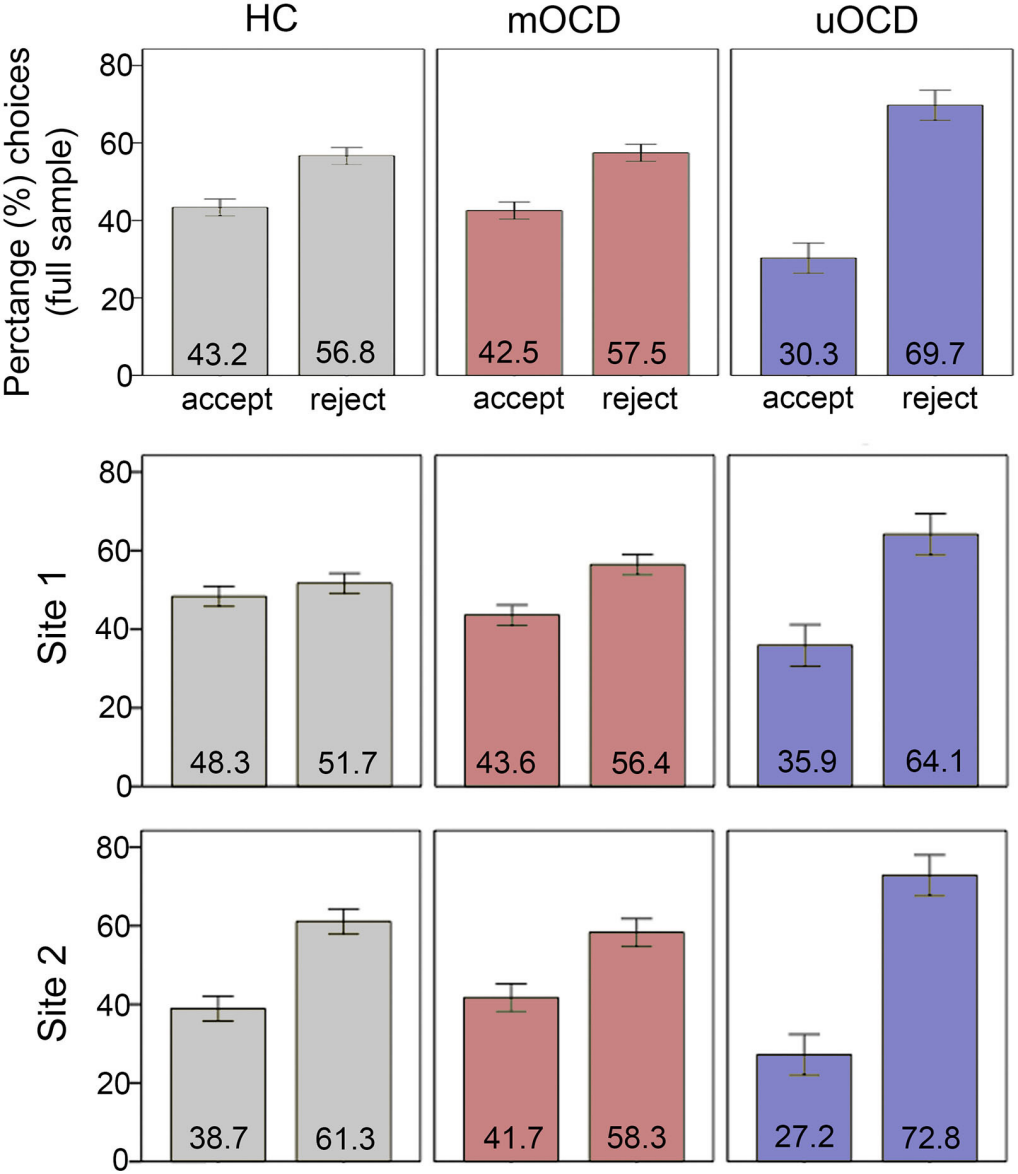
Percentage of gambles that were accepted vs. rejected in HC, medicated OCD (mOCD) patients, and unmedicated OCD (uOCD) patients in the full sample (top panel) and in each site separately. Numbers overlaid on bars represent group means. Error bars represent ±1 SEM.

Overall, participants accepted significantly fewer gambles (39.2%) than they rejected (60.8%). In the analysis of percentage of reject choices, there was a main effect of group (*F*_2,71_ = 4.6, *p* = 0.01), with uOCD patients rejecting significantly more gambles (accepting significantly fewer gambles) (percent reject for uOCD: 69.7%, SEM = 3.9) than mOCD patients (57.5%, SEM = 2.2, *t*_41_ = 2.9, *p* = 0.005, *d* = 0.92) and HCs (56.8%, SEM = 2.2, *t*_46_ = 3.1, *p* = 0.004, *d* = 0.94) (Figure 2). There was a main effect of study site (*F*_1,71_ = 4.6, *p* = 0.04, *d* = 0.56), with participants rejecting more gambles overall in Site 2 (62.6%, SEM = 2.3) than Site 1 (55.3%, SEM = 1.8). The interaction between group and site was not significant (*F*_2,71_ = 0.8, *p* = 0.45), indicating that uOCD patients’ propensity to reject more gambles (accept fewer gambles) than mOCD and HC was not different across sites.

**Figure 3:**
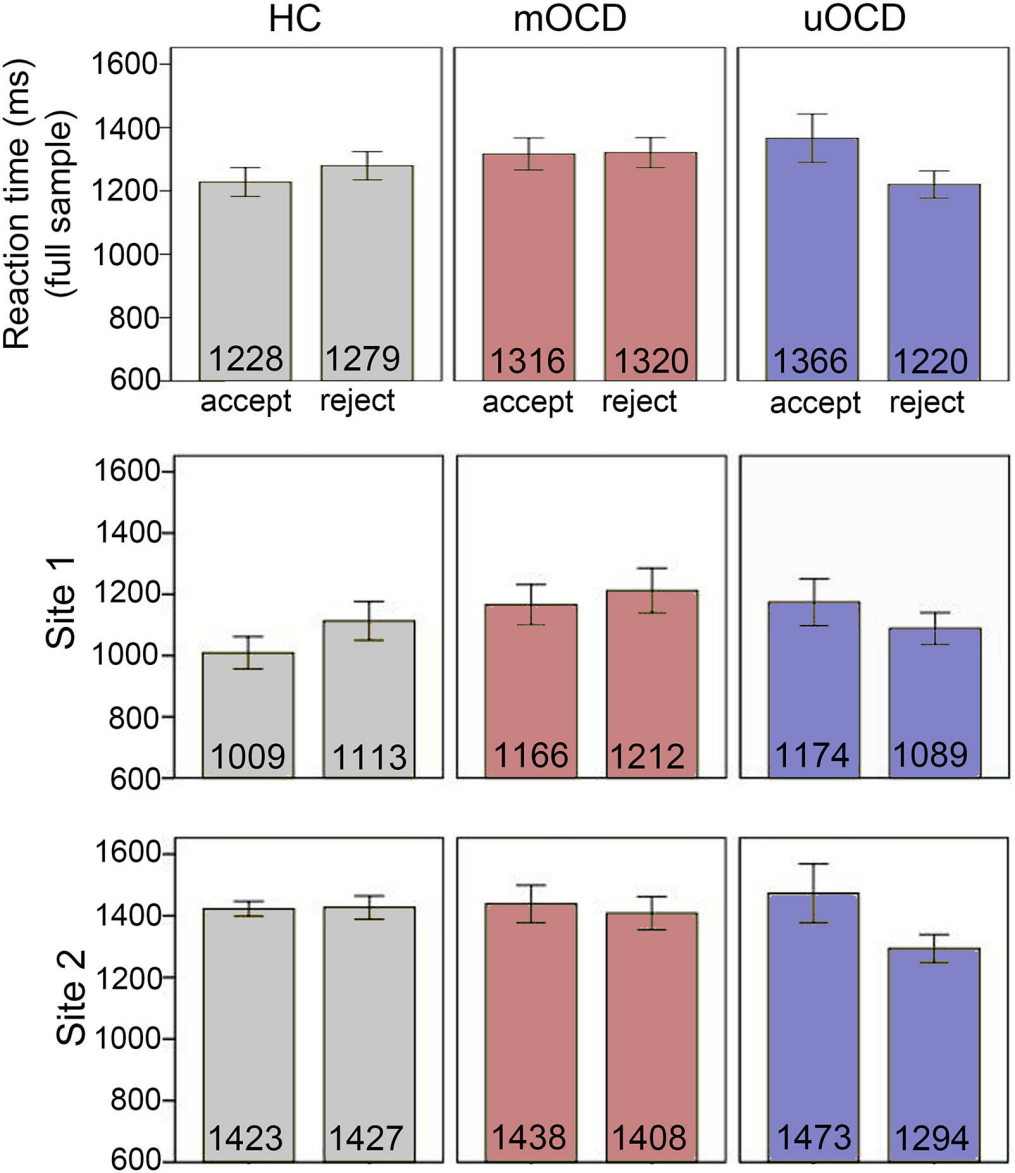
Reaction times to make choices in HC, medicated OCD (mOCD) patients, and unmedicated OCD (uOCD) patients in the full sample (top panel) and in each site separately. Numbers overlaid on bars represent group means. Error bars represent ±1 SEM.

For the analysis of decision times (RT to make choices) (Figure 3), there was an interaction between choice type (accept/reject) and group (*F*_2,71_ = 9.0, *p* < 0.001). Unmedicated OCD patients were significantly faster to reject (1,220 ms, SEM = 42.9) than accept (1,366 ms, SEM = 76.2) (*t*_13_ = 2.7, *p* = 0.02, *d* = 0.72), whereas HCs were significantly faster to accept (1,228 ms, SEM = 45.3) than reject (1,279 ms, SEM = 44.5) (*t*_33_ = 2.6, *p* = 0.02, *d* = 0.44) and mOCD patients showed no differences in RT between choices (1,316 ms, SEM = 50.7 and 1,320 ms, SEM = 47.2 for accept and reject choices, respectively, *t*_28_ = 0.2, *p* = 0.86). There was also a main effect of study site (*F*_1,71_ = 31.9, *p* < 0.001, *d* = 1.46): participants were faster overall to make choices in Site 1 (1120 ms, SEM = 38.8) than Site 2 (1416 ms, SEM = 26.6). This main effect of site was qualified by an interaction with choice type (*F*_1,71_ = 7.1, *p* = 0.01), indicating that the differences in RT between accept and reject choices was not the same for both sites. There was no three-way interaction between choice type, group, and site (*F*_2,71_ = 0.1, *p* = 0.94).

### Loss Aversion

**Figure 4:**
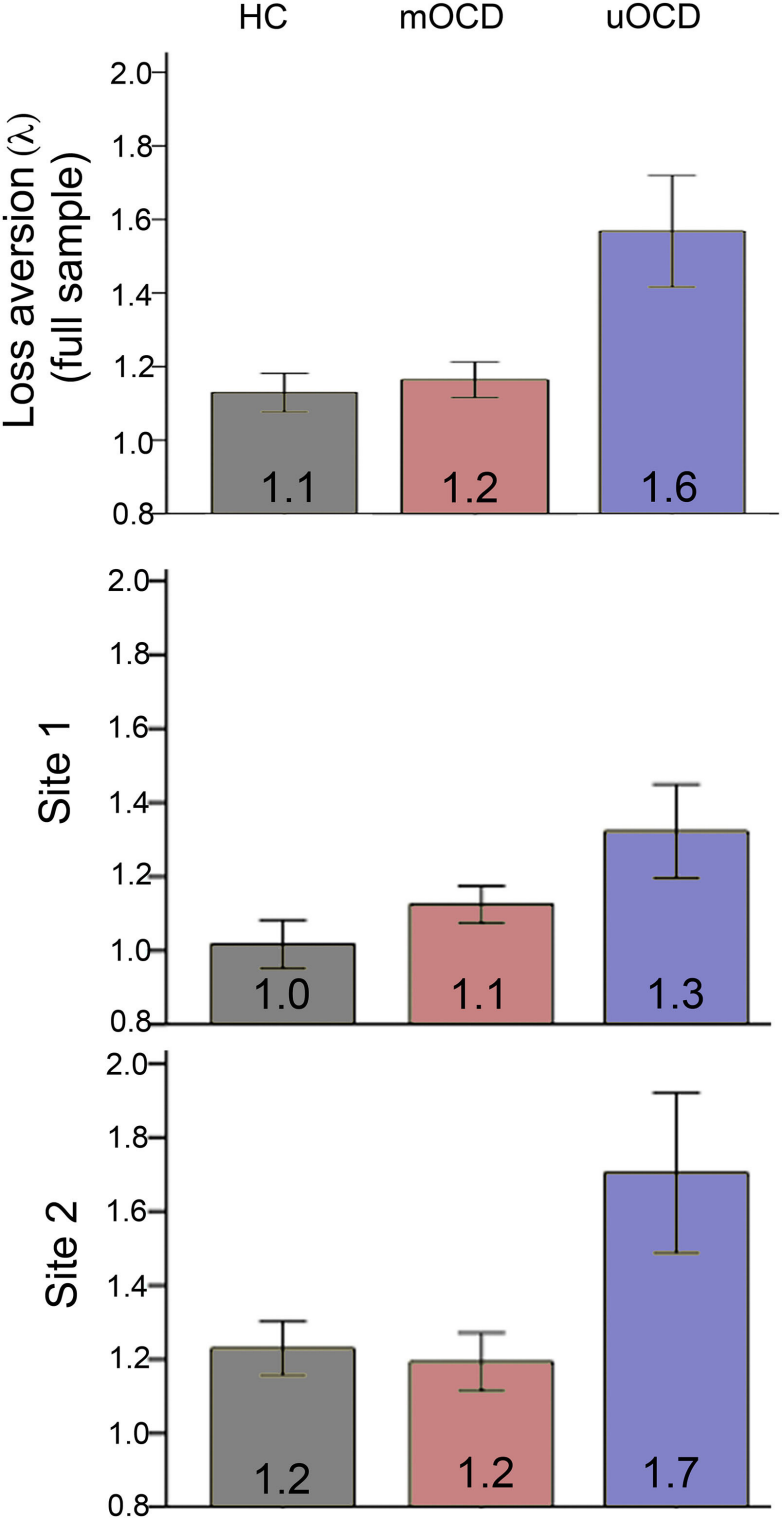
Loss aversion (λ = β_loss_/β_gain_) in HC, medicated OCD (mOCD) patients, and unmedicated OCD (uOCD) patients in the full sample (top panel) and in each site separately. Numbers overlaid on bars represent group means. Error bars represent ±1 SEM.

All three groups had loss aversion coefficients (λ) that were significantly greater than 1.0 (i.e., indicative of significant loss aversion) (uOCD: λ = 1.57, SEM = 0.16, *t*_13_ = 3.7, *p* = 0.002, *d* = 1.0; mOCD: λ = 1.16, SEM = 0.05, *t*_28_ = 3.4, *p* = 0.002, *d* = 0.62; HC: λ = 1.13, SEM = 0.05, *t*_33_ = 2.5, *p* = 0.02, *d* = 0.43). In the ANOVA comparing study site and group, there was a significant main effect of group (*F*_2,71_ = 6.4, *p* = 0.003, Figure 4): uOCD patients exhibited greater λ values than mOCD patients (*t*_41_ = 2.5, *p* = 0.02, *d* = 0.92) and HC (*t*_46_ = 3.5, *p* = 0.001, *d* = 0.96), who were not different from each other (*t*_61_ = 0.5, *p* = 0.65). Thus, not only were uOCD patients more likely to reject (less likely to accept) a gamble overall (Figure 2), the difference in slopes of the lines predicting rejection from loss value and acceptance from gain value was larger in the uOCD group compared to the other two groups. Note that the finding of increased λ is not equivalent to the finding of increased percentage of reject choices, because the λ analysis characterizes choices based on the change in loss/gain value, whereas the analysis of percentage reject choices does not take values into account.

**Figure 5:**
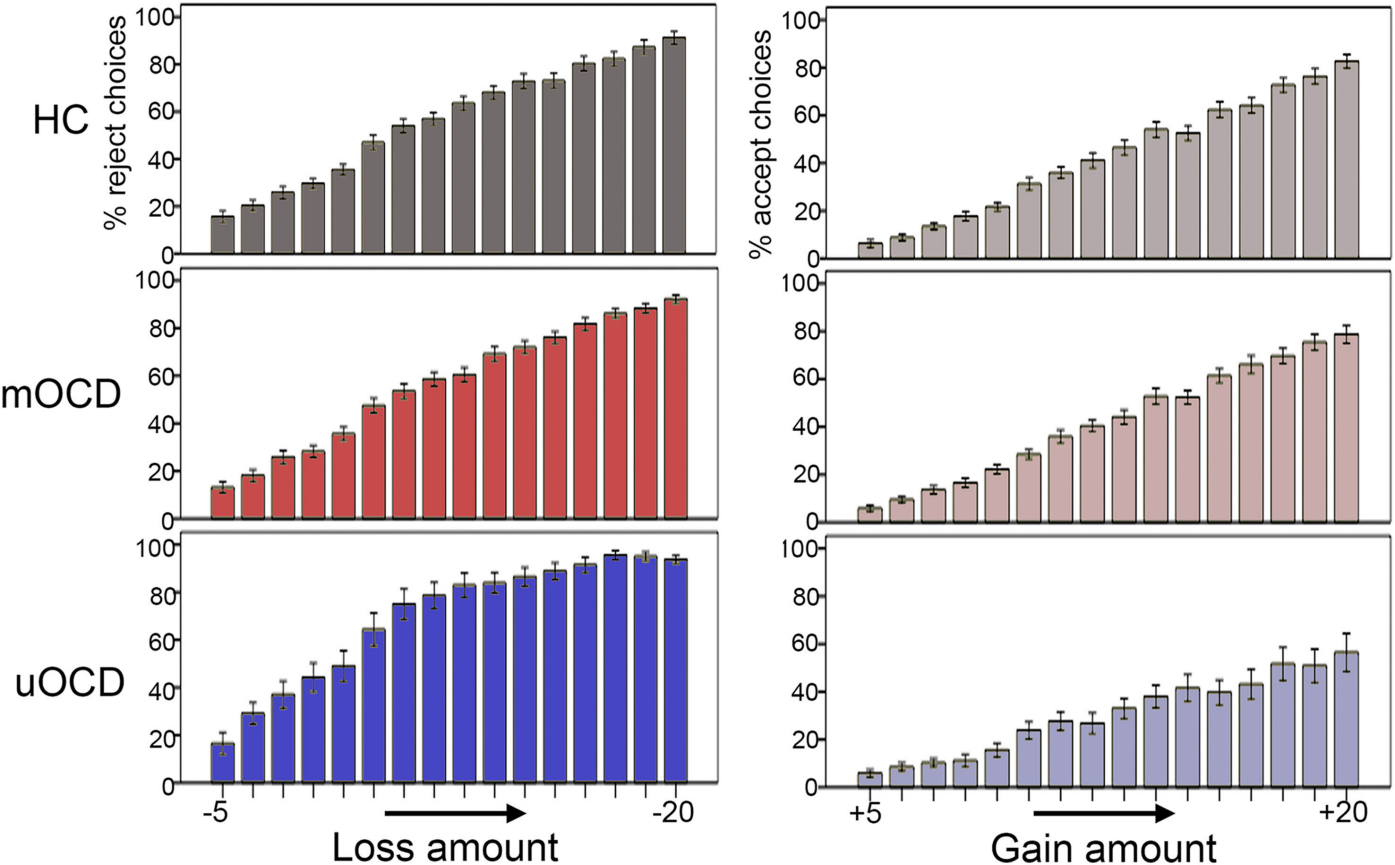
Percentage of gambles that were rejected in relation to increasing losses (−5 to −20 in one-point increments) (left panel) and percentage of gambles that were accepted in relation to increasing gains (+5 to +20 in one-point increments) (right panel) in HC, medicated OCD (mOCD), and unmedicated OCD (uOCD) in the full sample. Error bars represent ±1 SEM.

Greater λ values in uOCD appears to be driven by both a steeper increase in the likelihood to reject with increasing losses (Figure 5, left) as well as a flatter increase in the likelihood to accept with increasing gains (Figure 5, right). Note that although the λ coefficient is a relative measure between the loss and gain slopes, the pattern shown in Figure 5 need not necessarily be the case and that a greater λ could be hypothetically driven by a flatter slope for gains and no change in slope for losses, or a steeper slope for losses and no change in slope for gains.

There was also a main effect of study site on λ (*F*_1,71_ = 6.7, *p* = 0.01, *d* = 0.59), with participants in Site 2 showing greater λ values overall (λ = 1.32, SEM = 0.44) than those in Site 1 (λ = 1.10, SEM = 0.25) (Figure 4). The two-way interaction between site and group was not significant (*F*_2,71_ = 0.99, *p* = 0.38), indicating that λ was greater for uOCD patients at both sites. The consistency of the λ findings across sites was confirmed by examining the main effect of group (uOCD/mOCD/HC) on λ separately for Site 1 and Site 2. The significant effect of group on λ was replicated across sites (Site 1: *F*_2,31_ = 3.3, *p* = 0.05; Site 2: *F*_2,40_ = 5.3, *p* = 0.009). Analysis of log(λ) values revealed the same effects, with a main effect of group (*F*_2,71_ = 5.6, *p* = 0.005) and study site (*F*_1,71_ = 5.4, *p* = 0.02), but no interaction between factors.

### Disentangling the Contribution of Loss Aversion and Risk Aversion to λ

In our follow-up regression analysis, we examined the relationship between the likelihood of rejecting a gamble and the gamble’s gain/|loss| value for the subset of gambles where gain = |loss|, which allowed us to examine whether the effects of value on choice behavior were more consistent with a risk aversion-only model (which would predict no effect of value on behavior [parameter estimate not statistically different from zero] in this subset of gambles) or loss aversion-only model (which would predict a positive effect of value on behavior [parameter estimate statistically greater than zero] in this subject of gambles). There were significantly different parameter estimates for uOCD compared to HC (the reference) but not mOCD compared to HC. Overall, the parameter estimate for HC was not significantly different from zero (β = 0.03, *p* = 0.27), indicating that the likelihood of rejecting a gamble was not related to gain/|loss| values among this subset of gambles in the HC group. Medicated OCD patients were not significantly different from HC (β = 0.05, *p* = 0.27). By contrast, uOCD showed parameter estimates that were significantly greater than HC (β = 0.14, *p* = 0.02), indicating that as the gain/|loss| value of the gamble increased, uOCD were significantly more likely to reject the gamble. Overall, these data are consistent with our findings from the full sample of gambles showing an increased λ in uOCD, and importantly indicate that the λ effects cannot be explained by group differences in risk aversion only.

### Effects of Sex on Findings

Even though there was not a significant difference in the distribution of men vs. women between the three groups, women did make up a larger proportion of the uOCD group than the other groups. Given prior research suggesting that women are more loss and risk averse than men (21, 33, 34), we investigated whether sex could affect our results. We performed an exploratory analysis comparing men and women on the loss aversion λ within each of the groups using independent samples *t*-tests. There were no statistically significant differences between men and women in λ, although, numerically, in all three groups, women actually showed lower λ values than men (uOCD: men: λ = 1.9, SEM = 0.2, women: λ = 1.5, SEM = 0.1; mOCD: men: λ = 1.2, SEM = 0.9, women: λ = 1.1, SEM = 0.9; HC: men: λ = 1.2, SEM = 1.0, women: λ = 1.1, SEM = 0.8). When collapsing across mOCD and HC participants (where the numbers of males and females were relatively equal), an independent samples *t*-test examining sex differences in λ revealed a trend effect (*t*_61_ = 1.96, *p* = 0.06) whereby females (*n* = 34, λ = 1.1, SEM = 0.04) showed lower λ values than males (*n* = 29, λ = 1.2, SEM = 0.1). Critically, the group difference in λ between uOCD, mOCD, and HC remained significant in an ANCOVA specifying sex as a covariate (*F*_2,73_ = 10.1, *p* < 0.001, covariate-adjusted means, HC = 1.1, SEM = 0.1; mOCD = 1.2, SEM = 0.1; uOCD = 1.6, SEM = 0.1). The effects of group (*F*_2,70_ = 7.6, *p* = 0.001) and site (*F*_1,70_ = 5.1, *p* = 0.03) also remained significant in an ANCOVA using group and study site as factors and sex as a covariate. These data indicate that the greater loss aversion among uOCD was not likely due to the greater proportion of women comprising that group.

### Effects of Symptom Severity

Given that uOCD had higher symptom severity scores than mOCD patients, we conducted a *post hoc* comparison of λ just between uOCD and mOCD, controlling for Y-BOCS scores (and Site). Results indicated that uOCD still showed significantly higher λ values than mOCD even after statistically controlling for Y-BOCS scores (*F*_1,38_ = 5.86, *p* = 0.02).

Measures of trait anxiety were collected at both sites using the Spielberger State-Trait Anxiety Inventory (STAI) (35). Three participants did not complete the scale. Of the remaining participants (*n* = 74), there was no correlation between self-reported trait anxiety and λ (*r* = 0.11, *p* = 0.35). In ANCOVAs controlling for trait anxiety, group differences in λ remained highly significant in models that included (*F*_2,57_ = 7.5, *p* = 0.001) and omitted (*F*_2,70_ = 9.0, *p* < 0.001) study site as a factor. Unfortunately, we did not obtain any other symptom measures that were the same across the two sites. Both sites did acquire measures of depression severity, although Site 1 used the Beck Depression Inventory (BDI) (36) and Site 2 used the Hamilton Depression Rating Scale (HDRS) (37). In an exploratory analysis examining the correlation between λ and depression severity within each site separately, results indicated that neither the BDI score at Site 1 (*r* = −0.15, *p* = 0.40) nor the HDRS score at Site 2 (*r* = 0.12, *p* = 0.46) was correlated with λ. These data suggest that the results are not likely to be driven by differences in general anxiety or depression severity.

### Correlations among Variables

Within each group, Pearson’s correlations examined the relationships between decision times to accept and reject gambles separately with the percentage of gambles that were accepted as well as λ. Due to the small sample size of the uOCD group, analyses were repeated using non-parametric Spearman’s rank correlations. For mOCD patients and HCs, there were no significant relationships using either correlational analysis approach between decision times and percent accept choices or λ. Unmedicated OCD patients showed a negative correlation between the percentage of gambles that were accepted and RT when accepting gambles (Pearson’s *r* = −0.85, *p* < 0.001; Spearman rho = −0.85, *p* < 0.001), indicating that patients who accepted fewer gambles (rejected more gambles) had slower decision times when they did accept them. Similarly, there was a positive correlation between loss aversion λ and RT when accepting gambles (Pearson’s *r* = 0.78, *p* = 0.001, Spearman rho = 0.84, *p* < 0.001). These correlations remained significant when controlling for study site (partial correlation *r* = −0.86, *p* < 0.001 and *r* = 0.76, *p* = 0.003, respectively). RT when rejecting gambles was not correlated with percentage of gambles that were accepted or λ in uOCD. There were no correlations between the above behavioral variables and Y-BOCS scores within either the uOCD or mOCD groups separately or in the full patient sample.

Dosage information was available for 25 of the 27 mOCD patients who were taking SRIs. We computed fluoxetine equivalence dosages and correlated these values with behavioral and clinical variables. SRI dosage was negatively correlated with Y-BOCS (Pearson’s *r* = −0.46, *p* = 0.02, Spearman’s rho = −0.40, *p* = 0.05), indicating that higher dosage was associated with lower symptom severity as might be expected. Dosage was not correlated with any other behavioral variable.

## Discussion

This study found increased loss aversion among unmedicated OCD patients compared to medicated patients and healthy controls, an effect that was replicated across two study sites. The pattern of decision times to accept or reject gambles was also different between uOCD patients and the other two groups. While uOCD patients showed a “fast no” pattern of responding with faster RTs to reject than accept gambles, HC showed the opposite pattern (slower to reject than accept), consistent with a prior report in healthy adults (38). Furthermore, within uOCD patients, those with greater loss aversion were slower to accept gambles, providing a link between decision times and choices in the task. Our follow-up analysis of those gambles where gain = |loss| indicated that a risk aversion model alone could not explain uOCD patients’ behavior, which was characterized by a greater tendency to reject gambles with higher gain/|loss| values even when expected value was held equal on gain = |loss| trials (i.e., when there was an equal likelihood of gaining and losing the same value).

It is unclear why only the unmedicated OCD patients, but not the medicated patients, showed greater loss aversion and altered decision times compared to HC. Across both study sites, OCD symptom severity, as measured with the Y-BOCS, was higher for uOCD compared to mOCD groups; significant effects in unmedicated patients could have been driven by greater overall severity of the disorder. Arguing against this possibility, however, are our findings that Y-BOCS scores were not correlated with behavioral measures and the fact that uOCD patients showed greater loss aversion than mOCD even after statistically covarying for Y-BOCS scores. Indeed, the mOCD group had average Y-BOCS scores that were in the moderate-to-severe range (mean = 19.7), yet their decision-making in the task was nearly identical to that exhibited by HC. It is alternatively possible that differences between uOCD and mOCD reflect an ameliorating effect of medication on OCD-related loss aversion in the mOCD group in the absence of a similar reduction in other features of the disorder that contribute to overall Y-BOCS scores. This hypothesis remains speculative, however, because we did not directly investigate the effects of medication on changes in decision-making. Despite the uncertainty regarding the reason for the differences between uOCD and mOCD groups, our findings suggest that characterizing the full scope of impairment in OCD may require a variety of behavioral assessments. In particular, our data indicate that measures of decision-making could be used to identify subgroups with behavioral alterations that would not be identified by measures of overall symptom severity, but could nevertheless be targeted by treatment. Furthermore, the fact that we found behavioral differences using a task with monetary stimuli suggests an underlying dysfunction in loss processing that extends beyond patients’ symptoms. Overall, this finding adds to the body of work examining cognitive mechanisms of OCD and suggests that increased loss aversion, perhaps in combination with other proposed mechanisms including increased error processing, uncertainty and doubt, and impaired task switching and response inhibition [see Ref. (39) for a review], could be contributing to the complex phenotype of the disorder. As we unfortunately did not measure global functioning or quality of life, future work will be required to determine the functional consequences of increased loss aversion in OCD.

Few prior studies have examined loss aversion in patient populations. Patients with dementia (18), schizophrenia (13), and amygdala damage (20) show reduced loss aversion compared to healthy controls. Conversely, patients with depression exhibit increased loss aversion (9). It is not likely that the findings in the uOCD group are due to depression in our sample, however, as only two patients (one mOCD and one uOCD) had a comorbid diagnosis of major depression at the time of testing, and there was a greater prevalence of depression NOS and history of major depressive disorder (MDD) in the mOCD compared to the uOCD group (Table 1, comorbidity differences were not statistically significant). Instead, these data suggest that the loss aversion may be a transdiagnostic abnormality that is prevalent in both OCD and MDD. It is notable that uOCD displayed a flatter increase in the likelihood to accept a gamble with increasing gains (Figure 5, right), suggesting an insensitivity to potential reward that is consistent with neuroimaging work in MDD (40–42) and, to a lesser extent, OCD (43). Although it might be predicted that patients with anxiety disorders would also show increased loss aversion, previous studies did not find differences when comparing healthy and anxious adolescents or adults (10, 11), and we did not find a correlation between trait anxiety and loss aversion in our sample.

Although the key finding of increased loss aversion in uOCD patients was replicated across study sites, there were overall differences between the sites. Individuals rejected more gambles overall (irrespective of group) and showed greater loss aversion in Site 2 compared to Site 1. Furthermore, decision times were significantly slower for both accept and reject choices in Site 2. As described in the Materials and Methods, there were methodological differences between the two sites that are likely contributing to these effects. First, the experiment at Site 2 was conducted in an fMRI scanner, whereas the experiment at Site 1 was a behavior-only study, and it is possible that scanner-related factors including noise and discomfort could slow response times. Furthermore, following Tom et al. (25), in Site 2, participants provided confidence judgments regarding their decisions (weakly/strongly), which were then collapsed to create two levels (accept/reject) for analyses. It is probable that this feature of the design served to increase the amount of time it took participants to make decisions. With regard to effects on loss aversion, it is also possible that this feature increased the overall salience of choices, which may have altered the balance between gain/loss sensitivity. Critically, however, there were no interactions between group and study site for any behavioral variables, revealing the striking consistency of group differences despite variable implementations of the task.

It should be noted that in this study, the HC and mOCD patient groups showed average loss aversion coefficients of 1.1 and 1.2, respectively. Although both of these coefficients were significantly greater than 1, they are considerably lower than initial estimates derived from the study by Tversky and Kahneman (15). It is unclear why these participants in our study showed minimal loss aversion; however, recent work in healthy individuals has revealed a wide range of loss aversion coefficients, and our findings are consistent with some prior studies also reporting low estimates of loss aversion in a laboratory setting (21, 22, 29).

This study has several limitations that should be addressed in future work. First, our unmedicated OCD sample was relatively small, and there was a large sex imbalance in the group. Although our post hoc analyses indicated that greater loss aversion among unmedicated patients was not due to the increased prevalence of females in this group, future work to replicate this finding in a larger sample will equal numbers of males and females will be necessary. Furthermore, we did not obtain the same symptom measures at both sites other than the Y-BOCS and trait anxiety. Although depression severity (measured at both sites but with different scales) was not correlated with loss aversion within each site, future studies would be improved by controlling for depression severity and more generally selecting multiple symptom measures that are the same across all participants. On a clinical level, it is also unclear how increased loss aversion in unmedicated patients is related to real-world impairment, and future studies should seek to determine whether functional disability is associated with this altered choice behavior. In addition, the present paradigm did not include gain-only or loss-only gambles and thus could not measure the shape of the value function. Future research would benefit from presenting patients with a variety of gamble types (gain-only, loss-only, and mixed gain-loss) to allow a full estimation of the value function (15). The study also did not vary probabilities, and thus, this work cannot speak to whether probability weighting is also affected in OCD. Importantly, under prospect theory, there is no effect of weighting when using acceptability judgments with the same probabilities (50/50 gambles), thus any differential probability weighting across patients and controls would not have impacted our results. Nevertheless, it would be interesting to investigate the weighting function in OCD, particularly given a recent report of probability weighting abnormalities in a group of medicated and unmedicated patients (27).

In conclusion, we found increased loss aversion and altered decision times only in unmedicated OCD patients, effects that were consistent across two study sites. To our knowledge, this is the first study to investigate loss aversion in OCD, adding to the growing body of work investigating psychiatric disorders within an economic decision-making framework. These findings reveal an abnormality of decision-making in a subgroup of patients not taking medication, suggesting that future treatments could be aimed at modulating gain/loss processing during decision-making in this population.

## Ethics Statement

All participants gave written informed consent in accordance with the Declaration of Helsinki. The protocol was approved by the institutional review boards at both the Icahn School of Medicine at Mount Sinai and University of Michigan Medical School sites.

## Author Contributions

KS designed the study, ran subjects, conducted analyses, and contributed to the paper. RG designed the study, conducted analyses, and contributed to the paper. ST designed the study and contributed to the paper. ES designed the study, conducted analyses, and contributed to the paper.

## Conflict of Interest Statement

The authors declare that the research was conducted in the absence of any commercial or financial relationships that could be construed as a potential conflict of interest.
